# Hydrogen sulfide regulates hippocampal neuron excitability via S-sulfhydration of Kv2.1

**DOI:** 10.1038/s41598-021-87646-5

**Published:** 2021-04-14

**Authors:** Mark L. Dallas, Moza M. Al-Owais, Nishani T. Hettiarachchi, Matthew Scott Vandiver, Heledd H. Jarosz-Griffiths, Jason L. Scragg, John P. Boyle, Derek Steele, Chris Peers

**Affiliations:** 1 Reading School of Pharmacy, University of Reading, Reading, RG6 6UB UK; 2 Division of Cardiovascular and Diabetes Research, LIGHT, Faculty of Medicine and Health, University of Leeds, Leeds, LS2 9JT UK; 3 Department of Neuroscience, John’s Hopkins University School of Medicine, Baltimore, USA; 4 Leeds Institute of Rheumatic and Musculoskeletal Medicine, University of Leeds, Leeds, LS2 9JT UK; 5 School of Biomedical Sciences, Faculty of Biological Sciences, University of Leeds, Leeds, LS2 9JT UK

**Keywords:** Post-translational modifications, Ion channels in the nervous system, Neurophysiology

## Abstract

Hydrogen sulfide (H_2_S) is gaining interest as a mammalian signalling molecule with wide ranging effects. S-sulfhydration is one mechanism that is emerging as a key post translational modification through which H_2_S acts. Ion channels and neuronal receptors are key target proteins for S-sulfhydration and this can influence a range of neuronal functions. Voltage-gated K^+^ channels, including Kv2.1, are fundamental components of neuronal excitability. Here, we show that both recombinant and native rat Kv2.1 channels are inhibited by the H_2_S donors, NaHS and GYY4137. Biochemical investigations revealed that NaHS treatment leads to S-sulfhydration of the full length wild type Kv2.1 protein which was absent (as was functional regulation by H_2_S) in the C73A mutant form of the channel. Functional experiments utilising primary rat hippocampal neurons indicated that NaHS augments action potential firing and thereby increases neuronal excitability. These studies highlight an important role for H_2_S in shaping cellular excitability through S-sulfhydration of Kv2.1 at C73 within the central nervous system.

## Introduction

Current awareness of the biological roles of endogenous hydrogen sulfide (H_2_S) is expanding rapidly, as our understanding of its production and cellular targets continue to develop^1–3^. Enzymatic production of H_2_S from cysteine is regulated by cystathionine γ lyase (CSE) and cystathionine β synthetase (CBS). With reference to the central nervous system (CNS) 3-mercaptopyruvate sulfurtransferase (3MST) along with cysteine aminotransferase has also been demonstrated to generate H_2_S in the brain^4, 5^, with additional H_2_S protein-bound sulphur “stores”^6^. H_2_S levels in the brain have been reported in the µM range^7, 8^, however real time measurements are challenging given the physiochemical nature of the gasotransmitter^9–11^. While physiological levels are known to mediate diverse signalling cascades, there is the potential to develop cellular toxicity however a consensus on the molecular basis of H_2_S mediated neurotoxicity is lacking^12, 13^. Furthermore, some effects of H_2_S have been demonstrated to occur through polysulfides (H_2_S_n_) which are important physiological signalling species in themselves^14, 15^.

In the CNS, the gasotransmitter, H_2_S promotes induction of long-term potentiation^7^, modifies astrocytic Ca^2+^ signalling^16^ and protects neurons against oxidative stress^17^. Given the diversity of effects, it is perhaps surprising that one mechanism of action is emerging as vital in regulating these processes; the modification of protein thiol groups termed S-sulfhydration, in which the thiol –SH groups in cysteine residues are converted to persulfide –SSH groups^1, 18^, leading to modulation of protein function and/or protection against oxidation. Such a post-translational modification is analogous to the widespread S-nitrosylation of diverse proteins by nitric oxide^19^. Studies have suggested that up to 50% of cellular proteins may be basally S-sulfhydrated^1, 18^, implying that this post-translational modification is of widespread physiological importance.

Ion channels are a significant and expanding family of substrates for regulation by H_2_S^20^. Clear evidence for modulation of vascular K_ATP_ channels by S-sulfhydration has been presented^21^ and, given the numerous ion channels within the CNS that may be modulated by H_2_S^20^, it seems likely (although has yet to be demonstrated) that S-sulfhydration may be an important modulatory mechanism within the CNS. Voltage-gated potassium channels (Kv) are the most diverse ion channel family^22^ and some subfamilies have already been reported as substrates for H_2_S^23, 24^. In the present study, we have investigated the potential modulation of the delayed rectifier channel Kv2.1 by H_2_S. This channel is unique in that it is regulated through post translational modifications that have profound functional consequences. For example, phosphorylation of Kv2.1 dramatically alters excitability of central neurons, as has been demonstrated in hippocampal neurons^25–27^ suggesting that the mechanisms regulating these channels are highly important to signalling within the CNS.

## Experimental procedures

### HEK293 cell culture

Wild-type and Kv2.1-transfected HEK293 cells (the latter a gift from Dr JS Trimmer, Davis, CA) were maintained at 37 °C (95% air:5% CO_2_) in DMEM containing 2mM glutamine, 10% fetal bovine serum, 1% Glutamax and 1% penicillin/streptomycin^28^. The Kv2.1 gene (*KCNB1*) was originally cloned from rat. The C29A, C73A and C831A mutations were introduced into WT rat Kv2.1 using the Quik-Change Site-Directed Mutagenesis Kit (Stratagene, Cheadle, UK) according to the manufacturer’s instructions. All constructs were verified by DNA sequence analysis before transfection. Cells were plated either onto coverslips for electrophysiology or utilised for biochemical assays (see below).

### Primary neuron culture

Preparation of rodent neuronal cultures was carried out in accordance with local University of Leeds regulations, and reported in line with ARRIVE guidelines. Procedures were approved by the local University of Leeds ethics committee. Briefly, hippocampi from 6 to 8 day old Wistar rats were removed for mechanical and enzymatic dissociation as described before^28^. Tissue was incubated (15 min at 37 °C) in phosphate buffered saline containing 0.25 μg/ml trypsin (EC 4.4.21.4, from bovine pancreas; Sigma). Trypsin digestion was terminated by the addition of an equal volume of buffer containing 16 μg/ml soy bean trypsin inhibitor (SBTI, type I-S; Sigma), 0.5 μg/ml DNaseI (EC 3.1.21.1 type II from bovine pancreas; 125 kU/ml; Sigma) and 1.5 mM MgSO_4_. Following centrifugation (3000 rpm for 5 min), cells were resuspended in minimal Earle’s medium (MEM) supplemented with 10% fetal calf serum, 19 mM KCl, 13 mM glucose, 50 IU/ml penicillin and 50 μg/ml streptomycin. 100 μl of cell suspension was plated onto 10 mm diameter poly-l-lysine coated coverslips in a 24-well plate for electrophysiology. After 24 h, this medium was replaced with one containing 10% horse serum (in place of fetal calf serum) and 80 μM fluorodexoyurdine (FUDR) to prevent the proliferation of non-neuronal cells. By 48 h, this medium was exchanged for one containing Neurobasal media, supplemented with 2% B-27, 1% penicillin/streptomycin, 0.5 mM l-glutamine and 25 μM l-glutamic acid. Cells were maintained in a humidified incubator at 37 ºC (95% air: 5% CO_2_) for 7 days. All experiments were performed using cells cultured for 7 to 14 days.

### Electrophysiology

Fragments of coverslip with attached cells were transferred to a perfused (3–5 ml/min) recording chamber mounted on the stage of an Olympus CK40 inverted microscope. As previously described^28^, the standard perfusate for HEK293 cells (pH 7.2, 22 ± 1 °C) was composed of (in mM): NaCl (140), KCl (5), MgCl_2_ (2), HEPES (10), CaCl_2_ (2), and glucose (10). Patch pipettes had resistances 4–6 MΩ. Series resistance was monitored after breaking into the whole cell configuration throughout the duration of experiments. If a significant increase occurred (> 20%), the experiment was terminated. Series resistance was monitored and compensated for (60–80%) after breaking into the whole cell configuration throughout the duration of experiments. The pipette solution (pH 7.2) consisted of (in mM): KCl (140), EGTA (5), MgCl_2_ (2), CaCl_2_ (1), HEPES (10), and glucose (10). For some experiments an anti-Kv2.1 antibody (Neuromab, CA) was added to the intracellular solution (final concentration of 0.5 μg/ml). For HEK293 cells, two voltage protocols were adopted: (1) a series of depolarising steps from − 100 to + 80 mV in 10 mV increments for 500ms, (2) a single step to + 50 mV from − 70 mV for 100 ms at 0.2 Hz. For hippocampal neurons, similar protocols were used except for the inclusion of a single 30 ms prepulse to − 50 mV, to inactivate ‘A’-type potassium currents. Signals were sampled at 10 kHz and low pass filtered at 2 kHz. To investigate neuronal excitability, current-clamp recordings were undertaken. Action potential recordings were made in whole-cell current-clamp mode with signals low-pass filtered at 2 kHz and sampled at 10 kHz. Action potentials were evoked by 500 ms current pulses (0–200 pA). The perfusate consisted of (in mM): NaCl (135), KCl (5), CaCl_2_ (2.5), MgCl_2_ (1.2), HEPES (5), and glucose (10), and was bubbled with mixed gas (95% air/5% CO_2_; pH 7.4). Intracellular solution (pH 7.2) consisted of (in mM): KGluconate (110), NaCl (10), EGTA (11), Na_2_ATP (2), MgCl_2_ (2), CaCl_2_ (0.1), NaGTP (0.3), and HEPES (10). Voltage- and current-clamp analysis protocols were performed using an Axopatch 200A amplifier/Digidata 1200 interface controlled by Clampex 9.0 software (Molecular Devices, Foster City, CA). Offline analysis was performed using Clampfit 9.0 (Molecular Devices, Foster City, CA). Conductance values (G) were calculated as previously described^27^. Results are presented as means ± S.E.M., and statistical analysis performed using unpaired Student’s *t*-tests where *P* < 0.05 was considered statistically significant.

### Modified biotin switch assay

The assay was carried out as described previously^18^ but with some modifications: HEK293 cells expressing Kv2.1 WT or C73A mutant were cultured at 37 °C in a humidified atmosphere containing 95% air and 5% CO_2_ and treated with 250 µM NaHS for 1 h or 2 h when 90% confluent. Untreated cells were used as a controls. Cells were homogenized in non-denaturing lysis buffer (Tris-HCl 50 mM; NaCl 300 mM; EDTA 5 mM; Triton X-100 1%) unless stated otherwise all buffers were supplemented with complete cocktail protease inhibitors tablets (Life Science Roche, UK). Lysate was collected via centrifugation at 13,000 *g* (10 min at 4 °C). Protein concentration was determined using Pierce BCA Protein Assay Kit (Thermo Fisher Scientific, UK) according to the manufacturer’s instructions. To block free cysteines, lysates (250 μg total protein) were added to blocking buffer (HEPES 250 mM, pH 7.7; EDTA 1 mM plus 25% SDS and 20 mM methyl methanethiosulfonate [MMTS]) and incubated at 50 °C for 20 min with constant shaking at 1400 rpm. The samples were buffer exchanged and MMTS was removed using protein desalting spin columns (Thermo scientific, UK). Biotin-*N*-[6-(biotinamido)hexyl]-3′-(2′-pyridyldithio) propionamide (HPDP) was added to the buffer exchanged protein samples a to a final concentration of 4 mM and incubated in the dark for 90min at room temperature with constant shaking (1400 rpm), biotinylated proteins were then buffer exchanged using spin columns to remove biotin–HPDP in the neutralization buffer (HEPES 20 mM; NaCl 100 mM; EDTA 1 mM Triton X-100 0.5%, pH 7.7) and precipitated by streptavidin-agarose beads, for 16 h at 4 °C. The biotinylated proteins were eluted by SDS-PAGE sample buffer and subjected to Western blot analysis.

Anti-Kv2.1 antibodies K89/34 (NeuroMab, Davis, CA) were used to detect Kv2.1 WT and C73A. β-Actin was used as loading control (Sigma-Aldrich, UK). ImageJ software (NIH, Bethesda, USA) was used to analyse band intensities for Kv2.1 before and after the assay.

### Hydrogen sulfide generation

Hydrogen sulfide (H_2_S) was produced using the donor molecule sodium hydrosulfide (NaHS, Sigma-Aldrich, UK) and made up as a stock solution in Hank’s Balanced Salt Solution. This stock was then diluted in the perfusate to give the working concentration as stated in the text. To avoid degradation and loss of H_2_S the stocks were routinely made up immediately prior to experimentation. In addition, to confirm a role for H_2_S, the H_2_S-slow releasing molecule P-(4-methoxyphenyl)-p-4-morpholinylphosphinodithioic acid morpholine salt (GYY4137; Sigma-Aldrich, UK) was used. GYY4137 (1 M stock) was dissolved in DMSO and stored at − 80 °C, on the day of experiments the stock was diluted to 100 mM in perfusate and used on the same day, the final concentration of DMSO in the bath did not exceed 0.001%, when higher GYY4137 concentrations were used a 20 mM stock was made fresh in PBS and used on the same day for these experiments.

## Results

**Figure 1:**
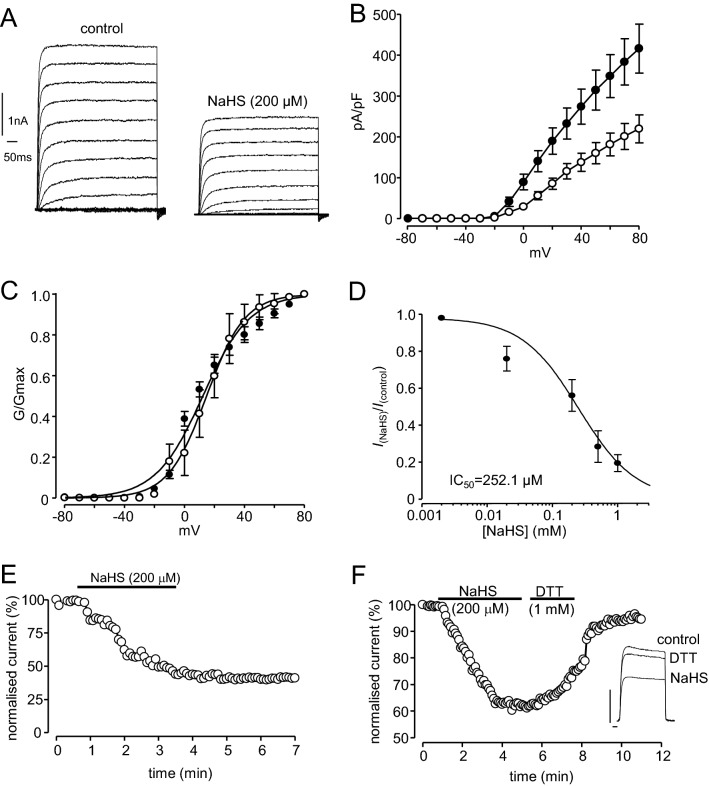
NaHS inhibits recombinant Kv2.1. (**A**) Families of currents evoked in a HEK293 cell stably expressing Kv2.1 before and during exposure to NaHS (200 μM). Currents were evoked by step-depolarizations, applied up to + 80 mV in 10 mV increments from a holding potential of − 70 mV. Scale bars apply to both families of currents. (**B**) Mean (± s.e.m.) current–density versus voltage relationships obtained in 17 cells before (solid circles) and during (open circles) exposure to NaHS (200 μM). (**C**) Mean (± s.e.m., each point taken from 5 to 7 cells) concentration–response relationship illustrating the effects of NaHS on Kv2.1. Inhibition was determined using currents evoked by step-depolarizations from − 70 to + 50 mV). (**D**) Plot of voltage-dependence of activation of Kv2.1 before (solid circles) and during (open circles) exposure to NaHS (200 μM). Data are means ± s.e.m. Curves were obtained by fitting data to the sigmoidal Boltzmann function. (**E**) Example time-series plot illustrating normalized current amplitudes evoked by step-depolarizations from − 70 to + 50 mV in a Kv2.1-expressing HEK293 cell. For the period indicated by the horizontal bar, NaHS (200 μM) was applied via the perfusate. (**F**) as (**E**), except that, following washout of NaHS, the cell was exposed to dithiothreitol (DTT, 1 mM) for the period indicated by the second horizontal bar. Inset shows example currents under the conditions indicated (DTT applied after washout of NaHS). Scale bars: 2 nA (vertical) and 10 ms (horizontal).

**Figure 2:**
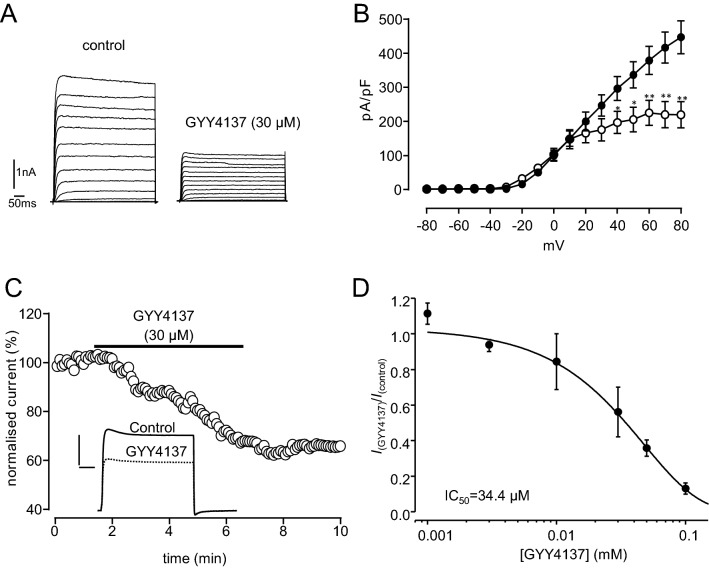
GYY4137 inhibits recombinant Kv2.1. (**A**) Families of currents evoked in a HEK293 cell stably expressing Kv2.1 before and during exposure to GYY4137 (30 μM). Currents were evoked by step-depolarizations, applied up to + 80 mV in 10 mV increments from a holding potential of -70 mV. Scale bars apply to both families of currents. (**B**) Mean (± s.e.m.) current–density versus voltage relationships obtained in 11 cells before (solid circles) and during (open circles) exposure to GYY4137 (30 μM). Significance: *P < 0.05; **P < 0.01 as compared with controls. (**C**) Example time-series plot illustrating normalized current amplitudes evoked by step-depolarizations in a Kv2.1-expressing HEK293 cell. For the period indicated by the horizontal bar, GYY4137 (30 µM) was applied via the perfusate, indicated by horizontal bar, followed by washout. Inset shows example currents under the different experimental conditions scale bars: 2 nA (vertical) and 10 ms (horizontal). (**D**) Mean (± s.e.m., each point taken from 3 to 5 cells) concentration–response relationship fitted to sigmoidal Boltzmann function illustrating the effects of GYY4137 on Kv2.1. Inhibition was determined using currents evoked by step-depolarizations from − 70 to + 50 mV.

To examine whether Kv2.1 could be regulated by H_2_S, we firstly recorded whole-cell currents from HEK293 cells stably expressing recombinant Kv2.1, as previously described^28^. Bath application of the H_2_S donor, NaHS (20 μM-1 mM), inhibited currents flowing through Kv2.1 channels in a concentration-dependent manner (Fig. 1A,B,C). Currents were inhibited at all activating test potentials (Fig. 1B) and there was no significant effect of NaHS on whole-cell conductance (Fig. 1C), suggesting that inhibition of current amplitudes was not attributable to altered gating. Inhibition of Kv2.1 currents was largely irreversible upon washout (e.g. Fig. 1E; 23.5 ± 4.1% recovery after 10 min, n = 12). However, current amplitudes could be recovered to near pre-NaHS treatment levels by exposure to the reducing agent dithiothreitol (DTT, 1 mM; Fig. 1F, 83.4 ± 3.7% recovery, n = 13). Similar results were obtained using another H_2_S donor molecule GYY4137 (Fig. 2). Kv2.1 currents at test potentials greater than + 40 mV by 30 µM GYY4137 (Fig. 2A,B), this concentration was determined following a dose-response relationship for the effects of the H_2_S donor GYY4137 (Fig. 2D). Normalized current amplitudes was inhibited by 40.1 ± 8.2%, n = 15, measured at + 50 mV and this inhibition was irreversible upon washout (Fig. 2C).

**Figure 3:**
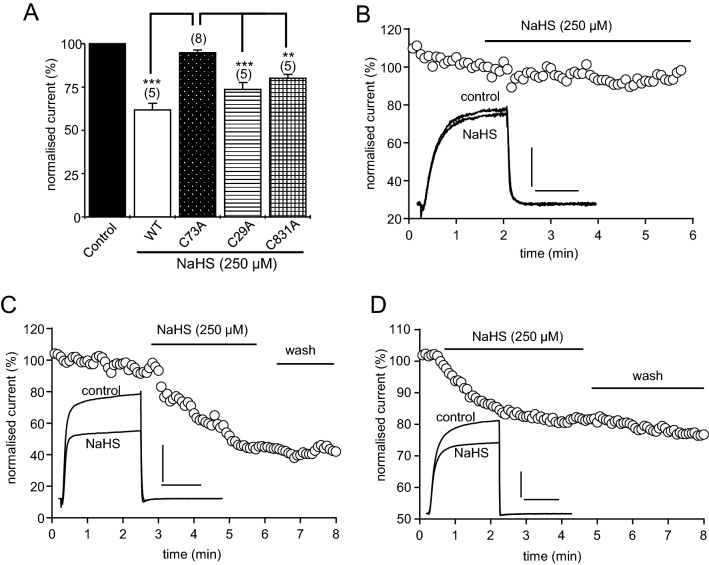
H_2_S does not inhibit Kv2.1 C73A but inhibit other recombinant mutant forms. (**A**) Bar graph showing normalised mean (± s.e.m.) effect of NaHS in wild-type (WT), C73A, C29A and C831A mutants of Kv2.1, as indicated (**indicates *P* < 0.01 and ***indicates *P* < 0.001 with the number of cells tested in parentheses). (**B**) Example time-series plot illustrating normalized current amplitudes evoked by step-depolarizations from − 70 to + 50 mV in a C73A Kv2.1-expressing HEK293 cell. For the period indicated by the horizontal bar, NaHS (250 µM) was applied via the perfusate. Inset shows example currents before and during NaHS application, as indicated. (**C**) Example time-series plot as in (**B**) for C29A Kv2.1-expressing HEK293 cell. For the period indicated by the horizontal bar, NaHS (250 µM) was applied via the perfusate, with irreversible effect upon washout for the time period indicated by the horizontal bar. Inset shows example currents before and during NaHS application for C29A, as indicated. (**D**) Same as in (**B** and **C**) except the example time-series plot illustrating a C831A Kv2.1-expressing HEK293 cell before and during application of NaHS (250 µM) for the period indicated by the horizontal bar, as in (**C**) the effect was irreversible upon washout, indicated by the horizontal bar. Inset shows example currents before and during NaHS application for C831A. Scale bars: 0.5 nA (vertical) and 50 ms (horizontal).

Reversal of H_2_S inhibition of Kv2.1 by DTT suggested a possible involvement of redox regulation of the channel protein. Sesti and colleagues^29^ have demonstrated that cysteine 73 is crucial to the response of this channel to oxidation, and so we generated a C73A mutant to explore the role of this residue in H_2_S regulation. As exemplified in Fig. 3A, and quantified in Fig. 3B, NaHS was without effect on the C73A mutant, whilst wild-type channels, studied in parallel, were significantly inhibited. Kv2.1 protein has an array of modifiable cysteine residues, some of which are not sensitive to oxidative modification^29^. To determine a role for other cystine residues in the observed NaHS effect, we generated several other mutant cell lines. C29A and C831A still provided functional channels however both cell lines were sensitive to inhibition following application of 250 μM NaHS (C831A by 20 ± 2% (n = 6); C29A by 26.4 ± 4% (n = 5), (Fig. 3C,D).

**Figure 4:**
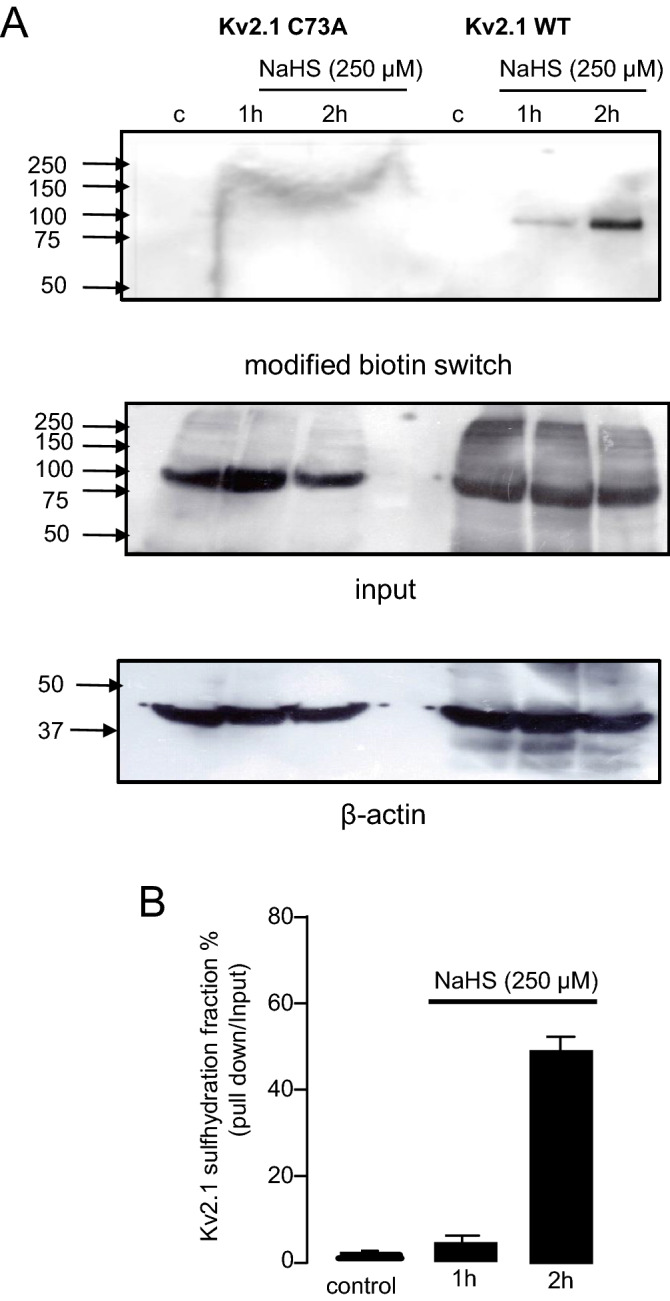
Kv2.1 sensitivity to NaHS is mediated by cysteine 73. (**A**) S-sulfhydration of WT but not C73A Kv2.1 by NaHS (250 µM) in HEK293 cells as detected by western blot analysis of biotinylated proteins produced by the modified biotin switch assay (upper bands). Middle bands demonstrate Kv2.1 protein input. Lower bands shows the loading control β-actin. Representative of five experimental repeats. (**B**) % fraction of S-sulfhydrated Kv2.1 following modified biotin switch assay over a 2 h period.

Many of the diverse effects of H_2_S in different systems arise due to specific, direct modulation of target proteins by S-sulfhydration (i.e. the conversion of –SH groups in cysteine residues into –SSH groups). S-sulfhydration is detectable via by the modified biotin switch assay^18^, and this approach was taken with the recombinant Kv2.1 protein. As illustrated in Fig. 4A, NaHS did indeed S-sulfhydrate recombinant Kv2.1, evident from 30 mins, suggesting that the mechanism of channel inhibition was due to direct protein modification (full blots can be found as Supplementary Figures S1, 2 online). This was supported by the observation that the C73A mutant form of the channel appeared resistant to this form of post-translational modification (Fig. 4A; full blots available as Supplementary Figure S3 online). To provide a level of quantification 50.8 ± 7.5%, (n = 3) of WT Kv2.1 channel protein (Fig. 4B) can be identified as S-sulfhydrated after 2 h treatment with NaHS (250 µM) following modified biotin switch assay (time dependence further explored in Supplementary Figure S4 online). The time discrepancies observed between functional and biochemical experimentation are likely to reflect technical limitations of single cell versus population studies. The combination of these methodologies has been extensively used when examining post-translational modifications of plasma membrane proteins.

**Figure 5:**
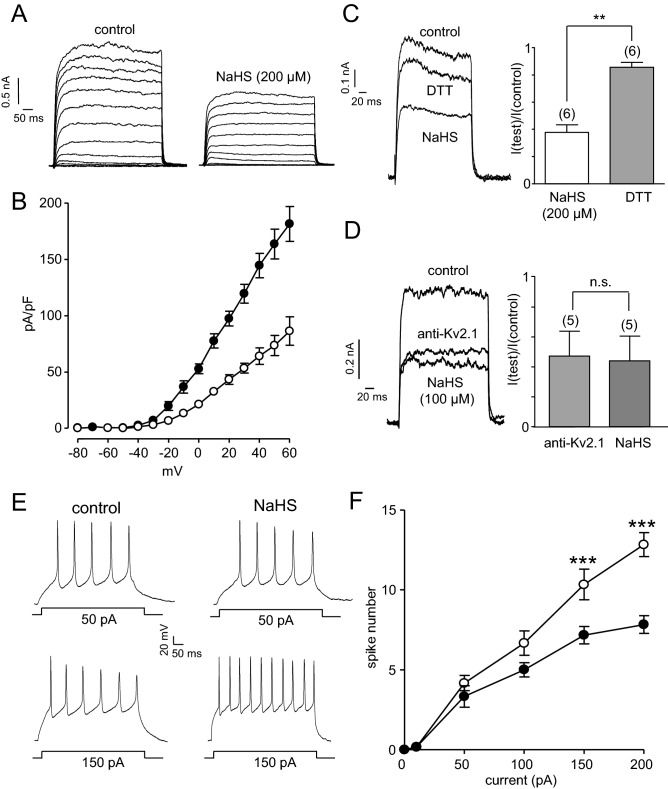
NaHS inhibits Kv2.1 and augments action potential firing in rat hippocampal neurons. (**A**) Families of currents evoked in a rat hippocampal neuron before and during exposure to NaHS (200 μM). Currents were evoked by step-depolarizations, applied up to + 60 mV in 10 mV increments from a holding potential of − 70 mV. Scale bars apply to both families of currents. (**B**) Mean (± s.e.m.) current–density versus voltage relationships obtained in 8 cells before (solid circles) and during (open circles) exposure to NaHS (200 μM). (**C**) Left, example currents evoked in the same neuron before (control) and during exposure to NaHS (200 μM), then during a subsequent exposure to DTT (1 mM) following washout of NaHS. Right, bar graph showing normalised mean (± s.e.m.) effect of NaHS and recovery of current amplitude by DTT. (**Indicates *P* < 0.01 with the number of cells tested in parentheses). (**D**) Left, example currents evoked in the same neuron immediately after obtaining the whole-cell configuration, after 10 min dialysis with an anti-Kv2.1 antibody, and then during a subsequent exposure to NaHS (100 μM), as indicated. Right, bar graph showing normalised mean (± s.e.m.) effect of the anti-Kv2.1 antibody, and the effects of NaHS following a minimum of 10 min dialysis in these cells. (n.s. indicates *P* > 0.05 with the number of cells employed indicated in parentheses). (**E**) Example voltage responses evoked by square wave depolarizing current injections (upper traces 50 pA, lower traces 150 pA) in hippocampal neurones perfused in the absence (left hand traces) or presence (right hand traces) of NaHS (200 μM). Scale bars apply to all traces. (**F**) Mean (± s.e.m.) number of evoked action potentials measured in response to 500 ms depolarizing current injections of varying amplitude. Action potentials were measured in the absence (solid circles) or presence (open circles) of 200 μM NaHS (***indicates *P* < 0.005, n = 6 for all current steps).

Kv2.1 is widely expressed in the CNS, and its ability to control the firing pattern of hippocampal neurons in particular has been described in detail (reviewed in Ref.^25^). As with HEK293 cells, we employed whole-cell patch-clamp recordings to demonstrate that NaHS caused significant inhibition of K^+^ currents in hippocampal rat neurons (Fig. 5A,B), at all activating test potentials studied. As was the case for recombinant Kv2.1, NaHS-mediated inhibition of K^+^ current amplitudes in hippocampal neurons could be recovered by DTT (1 mM; Fig. 5C).

Numerous different voltage-gated K^+^ currents contribute to the whole-cell K^+^ current in these cells. We and others have previously demonstrated that the contribution to the whole-cell current by Kv2.1 can be determined by intracellular dialysis (via the patch pipette) of an anti-Kv2.1 antibody^28, 30^. In agreement with our earlier studies, intracellular dialysis with this antibody reduced current amplitudes by around 50% (Fig. 5D). Subsequent exposures to NaHS (100 µM) did not significantly reduce currents further (Fig. 5D), indicating that NaHS was selective in its ability to inhibit Kv2.1 despite the presence of other voltage-gated K^+^ channels in hippocampal neurons.

Kv2.1 channel activity is a major determinant of hippocampal neuronal excitability, and can be dramatically modified, for example, by phosphorylation^25, 26^. To investigate whether H_2_S could alter hippocampal excitability, we employed current-clamp recordings. Application of NaHS caused a modest yet significant depolarization of cells (9.1 ± 1.8 mV, n = 6, *P* < 0.01), indicative of reported H_2_S effects on TRP channels in dorsal root ganglion neurons^31^. This effect was compensated by current injection, in order to examine the effects of depolarizing current injections in cells with a standardised membrane potential of − 70 mV (Fig. 5A,B). Square wave current injections evoked action potentials which increased in frequency with increasing current amplitude (Fig. 5A,B). In the presence of NaHS (200 µM), action potential frequency was augmented, particularly following larger current injections, as anticipated when Kv2.1 channels participate in neuronal excitability.

## Discussion

Our study identifies H_2_S as another important modulator of the voltage-gated K^+^ channel Kv2.1. This channel is highly expressed in the cortex and hippocampus where it is a major determinant of intrinsic neuronal excitability through a diverse complement of signalling cascades. High levels of constitutive phosphorylation determine the voltage-dependence of gating and location of Kv2.1, and both factors can influence neuronal excitability in a dynamic manner^25, 32, 33^. Activity-dependent, calcineurin-mediated dephosphorylation of Kv2.1 can suppress excitability^33^, and rapid rephosphorylation (e.g. via CDK5^34^) can restore excitability. However, this is dependent on the location of the phosphorylation site within the channel protein: for example, AMP-activated protein kinase-mediated phosphorylation at distinct residues (S440 and S537) can suppress excitability and exert hyperpolarizing shifts in the voltage-dependent activation, similar to calcineurin-mediated dephosphorylation of other residues^27^. In addition to alteration of gating via phosphorylation, Kv2.1 channel inhibition (e.g. following antisense knockdown) also increases high frequency excitability^35, 36^. The present study indicates that this phenomenon also occurs because of channel inhibition by H_2_S (Fig. 3). Thus, using larger depolarizing current injections, action potential frequency was augmented by H_2_S, suggesting that this gasotransmitter may influence excitability physiologically through modulation of Kv2.1 selectively. It remains to be determined if polysulfides mediated this inhibition as has been reported for Kv1.4 and Kv3.4^23^. This is likely to be mediated via S-sulfhydration of the channel given our biochemical evidence. There are time discrepancies between the acute exposure in the functional recordings and the biochemical observations, but these are comparable to other reports of ion channel post-translational modification (see Ref.^18^). This likely reflects the number of S-sulfhydrated channels required to produce a functional effect versus the amount of S-sulfhydrated protein detected via our biochemical experimentation.

Such complex regulation of a single channel target suggests it is of fundamental importance to the excitability of neurons in which it is expressed, but the fact that it is also a target for modulation by gasotransmitters supports the belief that it has even greater physiological significance. While evidence exists that other Kv channels are modulated by H_2_S, such as Kv7^24, 37, 38^, the neuronal localisation of these channels will ultimately determine their influence on action potential dynamics. For example, Kv7 activation via NaHS has been reported to suppress neuropathic pain^24^, indicative of a decrease in network excitability^39^. These studies have implicated the H_2_S in the mechanisms of neuropathic pain mediated by the Kv7 family. This furthers the diversity of ion channels targeted by H_2_S and also raises questions over selectivity^20^. With respect to the hippocampus, Kv7 channels has been localised to the axonal initial segment^40^ in contrast Kv2.1 channels display a predominant somatodendritic loci^41^. Therefore, discrete localisation of Kv channels^42, 43^ may underpin the respective action of H_2_S on neuronal output.

Kv2.1 has also been proposed to play a vital role in oxidative stress-induced apoptosis of central neurons: oxidants can initiate apoptosis in a Zn^2+^-dependent manner which leads to co-ordinated, Src kinase-mediated phosphorylation of the channel protein at an N-terminal tyrosine (Y124) together with p38 MAPK-mediated phosphorylation at a C-terminal site (S800). This in turn leads to rapid channel insertion into the plasma membrane; the resultant loss of cytosolic K^+^ triggers caspase activation and apoptosis. The present study suggests H_2_S may provide protection against oxidant-induced apoptosis via inhibition of Kv2.1. Indeed, we have previously shown that Kv2.1 is inhibited by carbon monoxide (CO), which is derived from heme oxygenases (HO-1 and HO-2). The inducible heme oxygenase, HO-1, is upregulated by numerous stress factors and its ability to provide protection against apoptosis in hippocampal neurons arises, at least in part, from the ability of its product, CO, to inhibit Kv2.1^28^. Interestingly, a similar Kv2.1-mediated protective mechanism may be active in some forms of cancer (in which resistance to apoptosis is a hallmark feature of the disease^44^) where HO-1 expression is constitutively high^45^.

Interestingly, research indicates that oxidative stress can modify Kv2.1 channels via a novel means, causing their oligomerization through formation of disulfide bridges with adjacent channel proteins. This in turn leads to channel clustering and promotion of apoptosis^29^, and it was proposed that this is an important aspect of both normal, age-related loss of neuronal function which is exacerbated in Alzheimer’s disease^29^. Animal studies also highlight modulation of the Kv2.1 protein function, to increase hippocampal neuronal excitability, in the 3xTg-AD model of Alzheimer’s disease^46^. In this regard, channel inhibition by H_2_S may be of particular importance: the modified biotin switch assay revealed that H_2_S caused S-sulfhydration of the channel protein (Fig. 2C), and this has been proposed to provide protection of vulnerable cysteine residues against oxidation^1^. This could have implications for cognitive decline and supports the use of H_2_S donors to modulate Alzheimer’s pathology^47–49^ however further consideration of the temporal profile of pathology alongside H_2_S administration is required.

In summary, we have shown that H_2_S inhibits both recombinant and native Kv2.1 and propose that this occurs via S-sulfhydration of the channel protein. This occurred in a concentration dependent manner; however, we cannot rule out the interactions of other proposed S-sulfhydrated molecular targets on our neuronal observations. However, consistent with the known physiological role of Kv2.1, we show that H_2_S-mediated inhibition contributes to augmentation of higher rates of evoked action potential frequencies with the Kv2.1 protein acting as a molecular rheostat^50^. This has implications for pathological conditions where (i) H_2_S production is altered (e.g. dementia^51^) or (ii) where the Kv2.1 protein has been oxidised at C73 (e.g. ageing^29^). Future studies will determine whether S-sulfhydration of Kv2.1 will provide protection against apoptosis arising from oxidative stress, for example, because of aging or neurodegenerative diseases.

## Supplementary information

Supplementary Informations.
