# Supplementary material for: Hydrogen sulfide regulates hippocampal neuron excitability via S-sulfhydration of Kv2.1
Source: Sci Rep. 2021 Apr 14;11:8194. doi: 10.1038/s41598-021-87646-5 (PMC8046973; doi:10.1038/s41598-021-87646-5)
Supplement: Supplementary file 1 — Supplementary Informations. [file 41598_2021_87646_MOESM1_ESM.pdf]

Supplementary Files

Hydrogen Sulfide regulates hippocampal neuron excitability via S-sulfhydration of Kv2.1

Dallas ML, Al-Owais MM, Hettiarachchi NT, Vandiver MS, Jarosz-Griffiths HH, Scragg JL, Boyle JP, Steele D, Peers C.

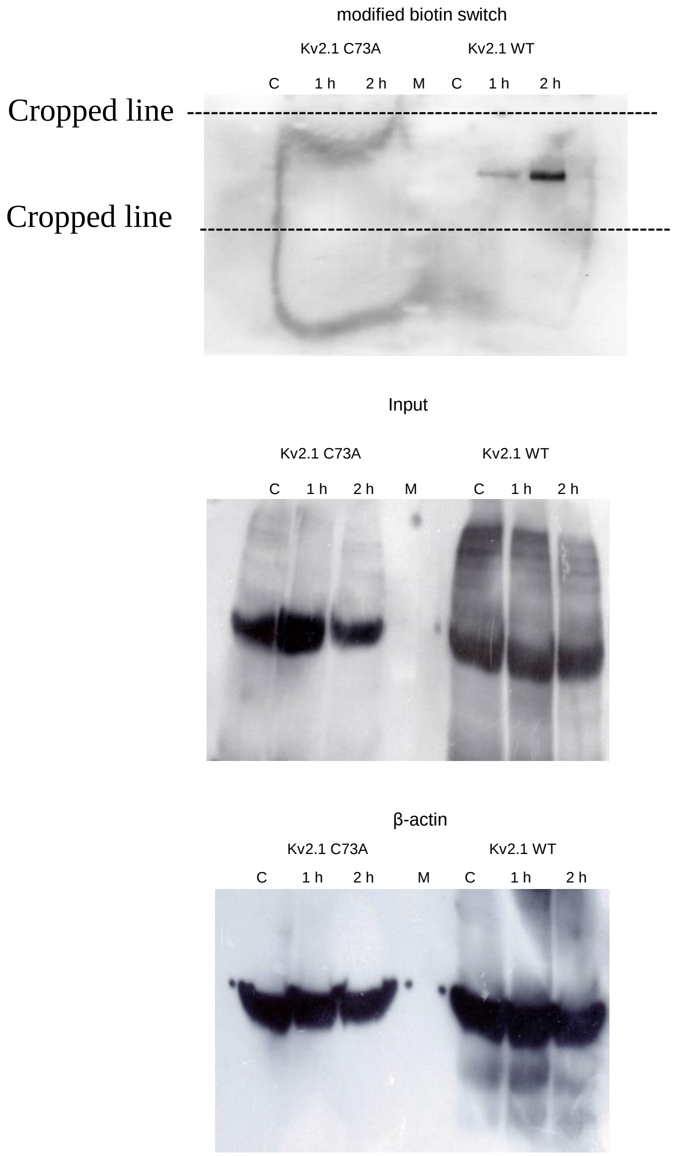

Supplementary Figure 1. Full Western Blots corresponding to Figure 4. Kv2.1 sensitivity to NaHS is mediated by cysteine 73. Full blots S-sulfhydration of WT but not C73A Kv2.1 by NaHS (250 μM) in HEK293 cells as detected by western blot analysis of biotinylated proteins produced by the modified biotin switch assay (upper bands, full blot scan with cropped line as indicated by dashed line). Middle bands demonstrate Kv2.1 protein input (full scan of separate blot). Lower bands shows the loading control β-actin (full scan of separate blot). All SDS-PAGE gels were loaded with samples to same order as shown on the heading of the blots. No corrections applied on the tone curve when images were scanned using a CanoScanLiDE25, however, input and β-actin images were resized to fit layout for the final used images.

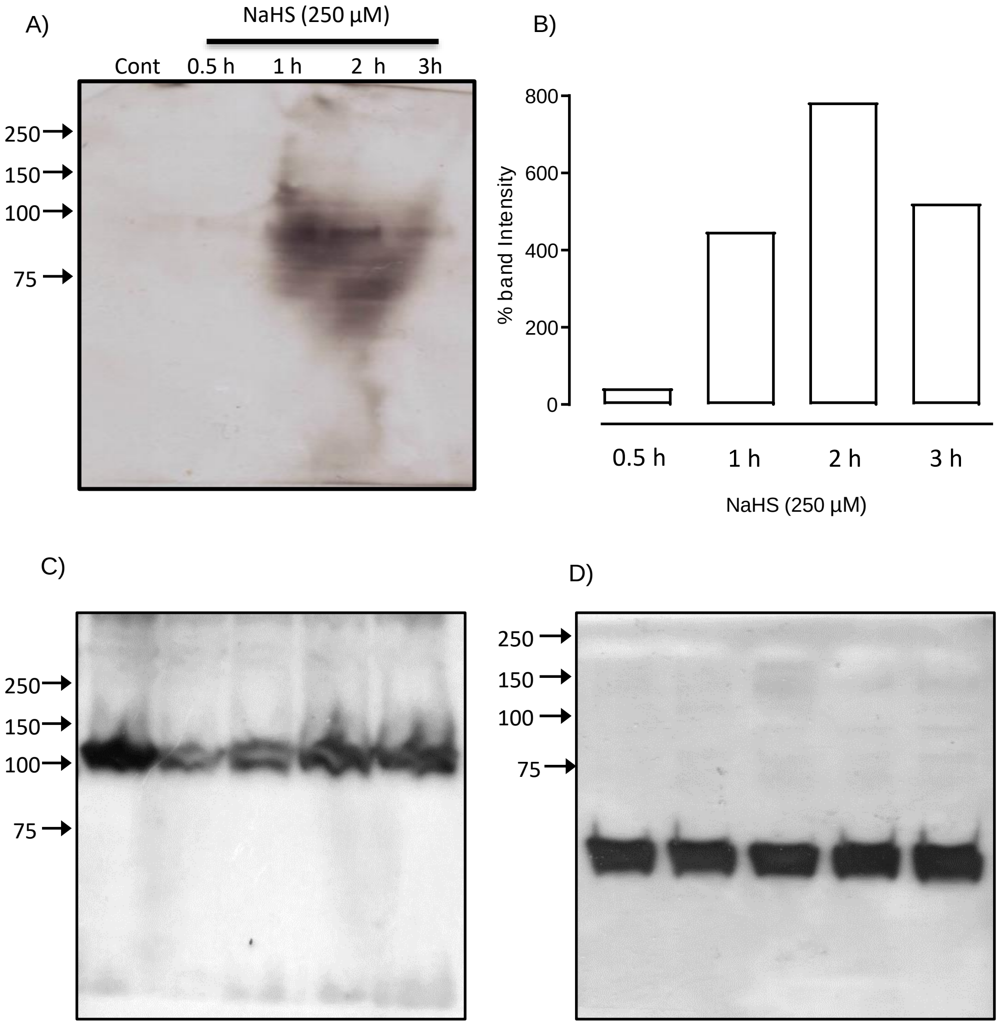

Supplementary Figure 2. S-sulfhydration time course of the wild type Kv2.1 by NaHS (250 µM) in HEK293 cells as detected by western blot analysis of biotinylated proteins produced by the modified biotin switch assay and following a pull down using streptavidin agarose beads. A) HEK cells expressing WT Kv2.1 were treated using NaHS (250 µM) at time points indicated, cells were then harvested and lysed in non denaturing lysis buffer as described in material and methods. B) Band intensity quantification was assessed using ImageJ processing program, all bands were normalised to the control (%), time point of 2 h was taken as the peak S-sulfhydration point and hence no other points were examined after that in subsequent experiments repeat. C) demonstrate Kv2.1 protein input and D) shows the loading control β-actin.

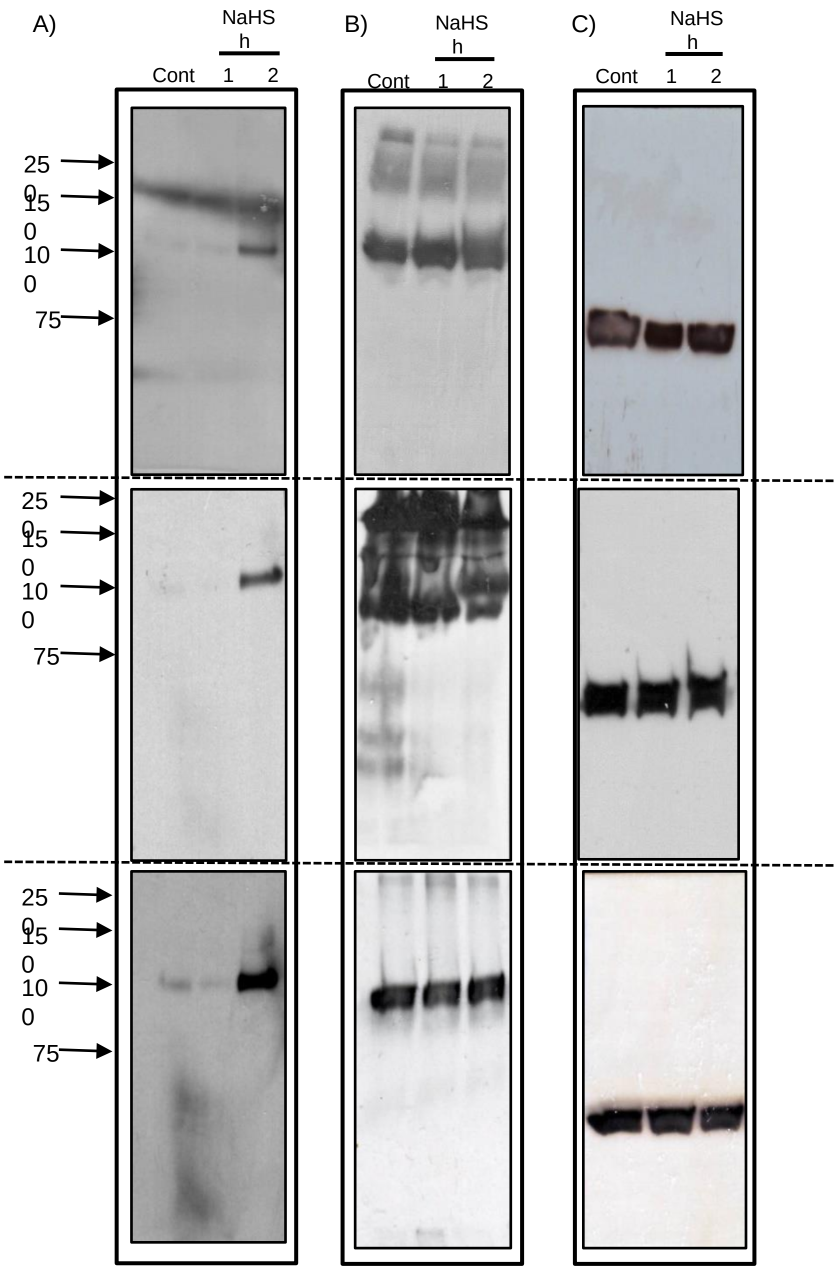

Supplementary Figure 3. Experiments repeat separated by dashed line of the wild type Kv2.1 S-sulfhydration using biotin switch assay; western blot analysis following modified biotin switch assay, A) demonstrated the WT Kv2.1 biotinylated proteins eluted from streptavidin agarose beads following a pull down at 1 h and 2 h treatment of HEK 293 cells expressing WT Kv2.1. Control indicate untreated HEK 293 expressing WT Kv2.1 B) shows WT Kv2.1 protein input and C) β-actin as loading control.

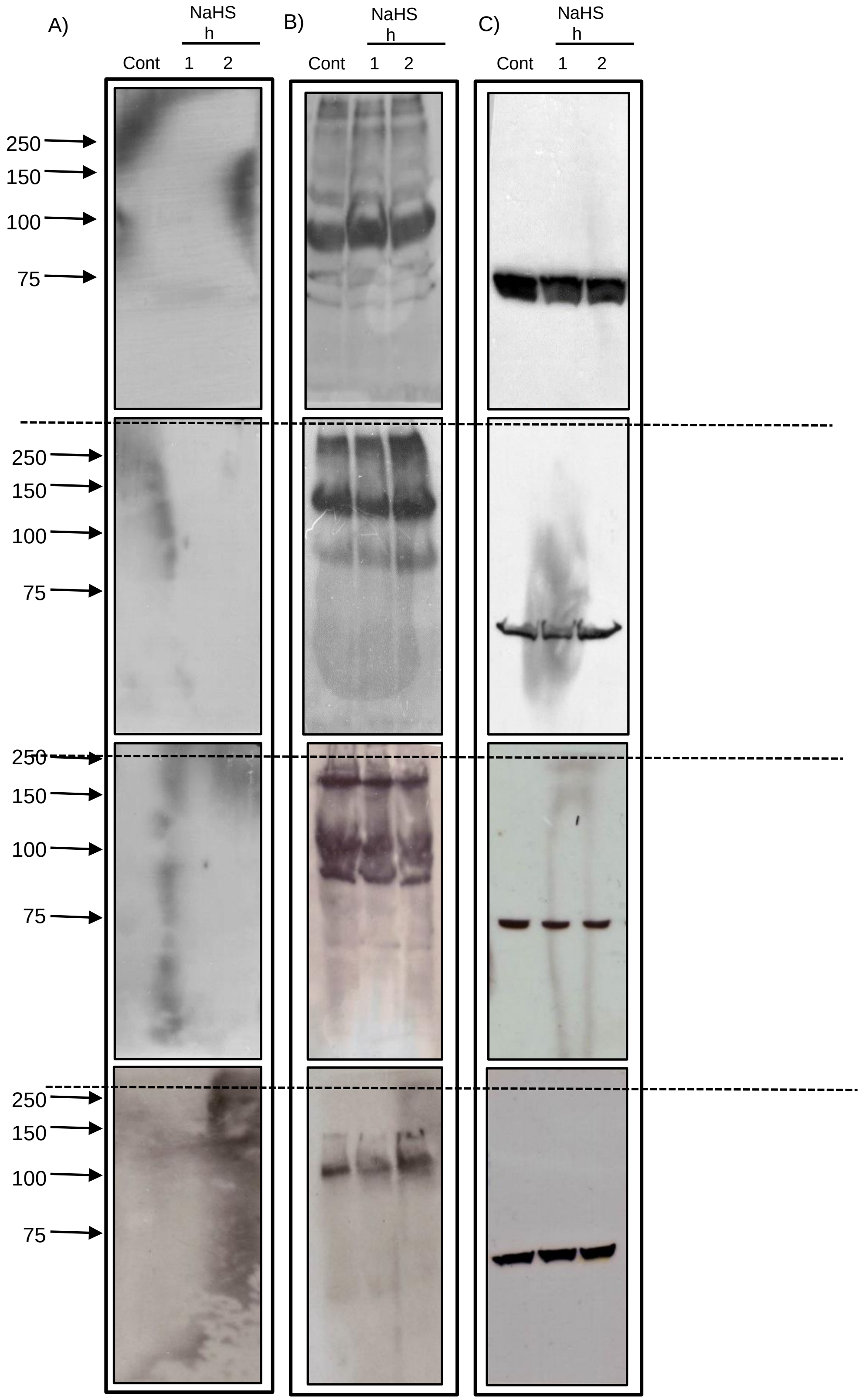

Supplementary Figure 4. S-sulfhydration was not detected in the Kv2.1 mutant C73A; biotin switch assay experiments repeat and western blot analysis, for details please see materials and methods section. A) biotinylated proteins eluted from streptavidin agarose beads following a pull down at 1 h and 2 h treatment with NaHS (250 µM) of HEK 293 cells expressing C73A Kv2.1. Sulfhydration was not detected at these time points in all experiments (dashed line). B) Detection of Kv2.1 C73A protein input and C) β-actin as loading control.
